# MINIMALLY INVASIVE SURGERY FOR GASTRIC CANCER: TIME TO CHANGE THE PARADIGM

**DOI:** 10.1590/0102-6720201600020013

**Published:** 2016

**Authors:** Leandro Cardoso BARCHI, Carlos Eduardos JACOB, Cláudio José Caldas BRESCIANI, Osmar Kenji YAGI, Donato Roberto MUCERINO, Fábio Pinatel LOPASSO, Marcelo MESTER, Ulysses RIBEIRO-JÚNIOR, André Roncon DIAS, Marcus Fernando Kodama Pertille RAMOS, Ivan CECCONELLO, Bruno ZILBERSTEIN

**Affiliations:** 1 Central Institute of Hospital de Clínicas; 2 Cancer Institute of the State of São Paulo, School of Medicine, University of São Paulo, São Paulo, SP, Brazil

**Keywords:** Gastric cancer, Minimally invasive surgery, Robotic gastrectomy, Laparoscopic gastrectomy, Stomach neoplasm

## Abstract

**Introduction:**

Minimally invasive surgery widely used to treat benign disorders of the digestive system, has become the focus of intense study in recent years in the field of surgical oncology. Since then, the experience with this kind of approach has grown, aiming to provide the same oncological outcomes and survival to conventional surgery. Regarding gastric cancer, surgery is still considered the only curative treatment, considering the extent of resection and lymphadenectomy performed. Conventional surgery remains the main modality performed worldwide. Notwithstanding, the role of the minimally invasive access is yet to be clarified.

**Objective:**

To evaluate and summarize the current status of minimally invasive resection of gastric cancer.

**Methods:**

A literature review was performed using Medline/PubMed, Cochrane Library and SciELO with the following headings: gastric cancer, minimally invasive surgery, robotic gastrectomy, laparoscopic gastrectomy, stomach cancer. The language used for the research was English.

**Results:**

28 articles were considered, including randomized controlled trials, meta-analyzes, prospective and retrospective cohort studies.

**Conclusion:**

Minimally invasive gastrectomy may be considered as a technical option in the treatment of early gastric cancer. As for advanced cancer, recent studies have demonstrated the safety and feasibility of the laparoscopic approach. Robotic gastrectomy will probably improve outcomes obtained with laparoscopy. However, high cost is still a barrier to its use on a large scale.

## RESUMO

**Introdução:**

A cirurgia minimamente invasiva amplamente usada para tratar doenças benignas do aparelho digestivo, tornou-se o foco de intenso estudo nos últimos anos no campo da oncologia cirúrgica. Desde então, a experiência com este tipo de abordagem tem crescido, com o objetivo de fornecer os mesmos resultados oncológicos e sobrevivência à cirurgia convencional. Em relação ao câncer gástrico, o tratamento cirúrgico ainda é considerado o único tratamento curativo, considerando a extensão da ressecção e linfadenectomia realizada. A gastrectomia convencional continua a ser a principal modalidade realizada em todo o mundo. Não obstante, o papel do acesso minimamente invasivo tem ainda de ser esclarecido.

**Objetivo:**

Avaliar e resumir o estado atual da ressecção minimamente invasiva do câncer gástrico.

**Método:**

Foi realizada revisão da literatura utilizando as bases Medline/PubMed, Cochrane Library e SciELO com os seguintes descritores: câncer gástrico, cirurgia minimamente invasiva, gastrectomia robótica, gastrectomia laparoscópica, neoplasia de estômago. A língua usada para a pesquisa foi o inglês.

**Resultados:**

Foram considerados para elaboração desta revisão 28 artigos, entre eles ensaios clínicos randomizados, metanálises, estudos coorte prospectivos e retrospectivos.

**Conclusão:**

A gastrectomia minimamente invasiva é opção técnica no tratamento do câncer gástrico precoce. Quanto ao câncer avançado, estudos recentes têm demonstrado a segurança e a viabilidade do acesso videolaparoscópico. A gastrectomia robótica provavelmente melhorará os resultados obtidos com a laparoscopia. Porém, o alto custo ainda é impedimento para sua utilização em larga escala.

## INTRODUCTION

The minimally invasive surgery widely used to treat benign disorders of the digestive system, has become the focus of intense study in recent years in the field of surgical oncology. However, the development and reproducibility of operations for the digestive tract cancer are still insufficient when compared to other specialties. Initially, the good results in colorectal cancer treatment by laparoscopic surgery encouraged other operations such as hepatectomy, esophagectomy, pancreatectomy, among others. Yet, some operations such as pancreatoduodenectomy and total gastrectomy with D2 lymphadenectomy still represent an important challenge because it demands great skill from the surgeon. More important than the traditional benefits of the laparoscopic approach as less pain, better cosmetic result, less tissue damage, shorter hospital stay and earlier return to work, is the apparent improved immune response to surgical trauma experienced by the patient. This might be one of the main reasons that has lead the development of laparoscopic surgery. Technological advances such as the robotic platform, with various forms of energy devices and improved optical systems and video, have proved to be useful in performing less traumatic procedures, better postoperative recovery and more adequate oncological results^24^. 

Since gastric cancer is very prevalent in the East, the pioneering laparoscopic operations were conducted there. The first laparoscopic gastrectomy dates from the early 1990s. Partial gastric resections were performed by Kitano in Japan in 1991 with Billroth I reconstruction and Goh in Singapore in 1992 with Billroth II. Azagra, in Belgium, performed the first total gastrectomy in 1995^3^ ^,^ ^10^.

Since then, the experience with this kind of approach has grown worldwide, aiming to provide the same oncological outcomes and survival to conventional surgery with the advantages of a less traumatic procedure. Therefore, minimally invasive surgery for early gastric cancer has been considered safe and an efficient alternative to open gastrectomy^26^ ^,^ ^27^. On the other hand, there is still no consensus regarding advanced gastric cancer, but many experienced surgeons have used this approach, reporting good short-term outcomes^4^ ^,^ ^22^.

The aim of this review was to evaluate and summarize the current status of minimally invasive resection of gastric cancer.

### MINIMALLY INVASIVE SURGERY FOR EARLY GASTRIC CANCER

Initially the laparoscopic approach was applied in study protocols in Japan and South Korea institutions. The authors described series with distal gastrectomy and limited lymphadenectomy, preferably in patients with early gastric cancer. Later on larger series studies were published. In 2009, Yakoub *et al.* published a systematic review on laparoscopic distal gastrectomy compared to the operations performed by laparotomy in early gastric cancer. The laparoscopic approach was associated with greater operative time and lesser pain, fever, blood loss, time to first walk and elimination of flatus, length of hospital stays and lower morbidity (10.5% vs. 20.1%). There were no differences in anastomotic complications. Patients operated by laparoscopy had fewer lymph nodes retrieved (mean difference of 4.6 lymph nodes). Nevertheless, there was no difference between the groups when the goal was a lymphadenectomy minor than D2^25^.

Kim *et al.* analyzed large series with 753 patients operated by laparoscopy. Early tumors were 78%. D2 lymphadenectomy was performed in 95% of cases with an average of retrieved lymph nodes of 34.1 and 40.6 for subtotal and total gastrectomy, respectively. Complications occurred in 9.2% and there was one death. With a mean of 56 months of follow-up, 5-year overall survival and disease free survival were greater than 96%^9^.

Vinuela *et al.* published a meta-analysis of randomized and nonrandomized studies including 3,055 patients and compared laparoscopic and open gastrectomy in gastric cancer patients (1,658 and 1,397, respectively). Stage I patients were 83%. Although mortality was similar, the laparoscopic approach was associated with lower morbidity, longer operative time, less blood loss, shorter hospital stays and higher number of retrieved lymph nodes by 3.9 nodes in the open gastrectomy group (p<0.001). However, the proportion of patients with less than 15 harvested nodes was similar between the groups (p=0.09), what could be enough for some western countries, but certainly not for eastern countries that recommend at least 25 lymph nodes retrieved for a standard D2 lymphadenectomy^23^.

To clarify this issue, two multicenter prospective randomized phase III trials are underway comparing open distal gastrectomy with laparoscopic distal gastrectomy for stage I gastric cancer. The KLASS 01 (Korean Laparoscopic Gastrointestinal Surgery Study) began recruitment in 2006 and finished in 2010. There were 1,415 patients operated (704 laparoscopic and 711 open) staging cT1N0M0, cT1N1M0, and cT2aN0M0. The procedures were performed by surgeons with extensive experience in laparoscopic gastrectomy (at least 50 cases/year). The primary endpoint was to evaluate 5-year overall survival, and secondary endpoints were to analyze the disease free survival, quality of life, morbidity and mortality, inflammatory and immune response and costs^8^. Preliminary results with 342 patients demonstrated no statistical difference in morbidity and mortality between the groups^7^. Another multicenter prospective randomized trial underway in Japan is the JCOG 0912 (Japan Clinical Oncology Group), which has as its primary objective to demonstrate non-inferiority of laparoscopic gastrectomy compared to open gastrectomy in early gastric cancer patients. Nine hundred and twenty patients stage IA (T1N0) or IB [T1N1 or T2 (MP) N0] were included. The interim results demonstrated benefits of the laparoscopic approach without jeopardizing survival. In the next months the final results of these two randomized trials will be presented, which probably will confirm laparoscopic distal gastrectomy as a treatment alternative for stage I patients^15^.

### MINIMALLY INVASIVE SURGERY FOR ADVANCED GASTRIC CANCER

As for early gastric cancer there seems to be consensus on the feasibility and oncology surgical quality, reports concerning the minimally invasive surgery for advanced cases have been published in recent years. This has paramount importance to the West, where the advanced cases rates exceed 80%. 

Lee and Kim analyzed 106 cases of laparoscopic gastrectomy for advanced cases with D2 lymphadenectomy with an average of 34 lymph nodes resected. The analysis of overall survival and disease free survival for each stage was similar to historical results treated by laparotomy^12^.

Cai *et al*. conducted a randomized prospective trial comparing 49 patients operated by laparoscopy and 47 by laparotomy, all with advanced disease and submitted to D2 lymphadenectomy. After 22 months of follow-up, there was no difference in survival between the two groups ^2^.

Martinez-Ramos *et al.* have published interesting meta-analysis, which analyzed the results of studies comparing open and laparoscopic gastrectomy. The minimally invasive approach was associated with shorter hospital stay, less blood loss and longer operative time. Moreover, there were no significant differences between the two groups concerning the number of dissected lymph nodes and no significant differences for cancer-related mortality risk (adjusted for 60 months of follow-up), although there was a tendency toward a protective effect for laparoscopic gastrectomy^14^.

Park *et al.* reported long-term results of a multicenter study (10 hospitals in South Korea, 239 patients) between 1998 and 2005. Extended lymphadenectomy (D2) was performed in 68% of cases and the average number of retrieved lymph nodes was 33.6. The mortality rate was 0.8 and the main complications were: wound infection (5%), bleeding (1.7%), fistula (1.7%) and pulmonary complications (0.8%). Overall complications rate was 15.9%. Overall survival and disease free survival at five years were 78.8% and 85.6%, respectively. These authors reported that survival by stage were similar to historical controls operated by laparotomy. Prognostic factors analysis showed that age, depth of invasion in the gastric wall (T parameter) and lymph node status (N parameter) were statistically significant in multivariate analysis^17^.

In 2012, Shinohara *et al.* published a study which analyzed the laparoscopic approach in advanced cases between 1998 and 2008. During this period laparoscopic gastrectomy increased from about 30% to almost 100% of the operated cases. Extended lymphadenectomy was performed in 39% vs 69% treated by laparotomy. Although mortality was similar in both groups, complications rate was lower in the laparoscopic group, where there were fewer lymph nodes retrieved, shorter operative time, less blood loss and shorter hospital stay^18^.

Recently, three randomized multicenter prospective trials have begun to survey the feasibility of minimally invasive surgery for advanced gastric cancer in South Korea, Japan and China. Probably, they will provide solid evidence regarding the use of this method in advanced stages patients (Table 1). 

**TABLE 1:** Randomized, multicenter, prospective controlled trials of laparoscopic gastrectomy in advanced gastric cancer treatment

	TRIAL	FASE	INCLUSION CRITERIA	PATIENTS (N)	PRIMARY ENDPOINT	SECONDARY ENDPOINTS	STATUS
JAPAN	JLSSG 0901	II/III	cN0-2 (excluding bulky N2)	500	Incidence of anastomotic leakage or pancreatic fistula; Relapse-free survival	OS; Proportion of LDG completion; Proportion of conversion to open surgery; Adverse events; Short-term clinical outcomes; Number of retrieved LN; Recurrence sites	Recruting
KOREIA	KLASS-02	III	cT2/cT3/ cT4a cN0-1 (including LN#7)	1,056	3-yr DFS	Early postoperative complication; Postoperative mortality; Late postoperative complication; Postoperative recovery index; Postoperative QOL; 3-yr OS	Recruting finished
CHINA	CLASS-01	III	cN0-3 (except bulky LNs)	1,050	3-yr DFS	Morbidity and mortality; 3-yr OS; 3-yr recurrence pattern; Postoperative recovery course; Inflammatory and immune response	Enrollment completed

KLASS=Korean Laparoscopic Gastrointestinal Surgery Study; JLSSG=Japanese Laparoscopic Gastric Surgery Study Group; CLASS=Chinese Laparoscopic Gastrointestinal Surgery Study; LDG= laparoscopic distal gastrectomy; ODG=open distal gastrectomy; LN=lymph node; OS=overall survival; DFS=disease free survival; QOL=qualite of life

In respect of total gastrectomy in advanced cases, few series were reported, probably due to great technical difficulty in the digestive tract reconstruction performed by laparoscopy. Lee *et al.* analyzed 94 cases of total gastrectomy with D2 lymphadenectomy. Complications were reported in 42.6% of patients and 9.6% were grade III (Clavien-Dindo)^11^. Zilberstein *et al.* reported 21 cases of D2 total gastrectomy with total laparoscopic digestive tract reconstruction with lateral esophagojejunal anastomosis with linear stapler, with only one anastomotic leakage treated conservatively. Operative time was similar to historical series, as well as the number of lymph nodes removed^29^.

 Oncological laparoscopic gastrectomy has a long learning curve. The surgeon should be well familiar with open gastrectomy as well as extended lymphadenectomy and have great experience with minimally invasive surgery, including endo-sutures. Hu *et al.* reported the need of, at least, 40 cases to improve the surgical time, reduce blood loss and increase the number of resected lymph nodes^5^.

### MINIMALLY INVASIVE SURGERY FOR GASTRIC CANCER IN BRAZIL

The first laparoscopic gastrectomy in Brazil was performed by Tinocco *et al.* in 1993. Over two decades after this report, the minimally invasive approach in gastric cancer treatment has not been routinely used and only few centers perform this treatment modality. There are many causes such as high costs of laparoscopic surgery for the public health system, high government taxes on imported materials (endostaplers, energy devices, etc.), difficulty to teach and promulgate this method and decentralization in the oncological diseases treatment. This pioneering group reported 92 cases of laparoscopic gastrectomy performed between 1993 and 2008, with 7.6% conversion rate, 14.1% morbidity and 5.4% mortality. The number of lymph nodes resected ranged from 21 to 57, with a mean operative time of 162 minutes. According to the authors, this kind of approach is safe and effective, nevertheless demands long learning curve^20^.

Oliveira *et al.* published a retrospective study comparing laparoscopic total gastrectomy with laparotomy for gastric cancer. Between 2009 and 2013, 111 patients stage I, II and III were operated. Conventional surgery was performed in 64 (57.7%) and 47 (42.3%) received treatment by laparoscopy, all with extended lymphadenectomy. There was no significant difference between the groups regarding age, gender, ASA (American Society of Anesthesiologists), tumor stage, need for blood transfusion, Bormann classification, negative margins, complications and mortality. Operative time and oral and enteral intake period were shorter in the laparoscopy group comparing to conventional technique. Yet, the average number of resected lymph node was 29.1 in the laparoscopy group and 35.1 in the conventional group (p=0.014). These short-term results have shown the benefit of minimally invasive surgery over conventional surgery^16^.

The Stomach, Duodenum and Small Intestine Unit of Hospital das Clínicas - University of São Paulo School of Medicine has also been pioneer in Brazil concerning the minimally invasive gastric resection for gastric cancer. Zilberstein *et al.* published the realization of laparoscopic gastrectomy with extended lymphadenectomy in 70 patients between 2007 and 2013. Most patients were submitted to partial gastrectomy (70%), with at least 37 lymph nodes removed. There was no conversion to open surgery and no mortality. As complications, there was only one esophagojejunostomy leakage and two injury of middle colic vessels, requiring partial colectomy^27^.

### ROBOTIC GASTRECTOMY FOR GASTRIC CANCER

Despite the benefit to patients obtained by the laparoscopic approach, it is known that this pathway often causes greater physical effort and wear of the medical team, in addition to other drawbacks such as, for example, 2D view. In this regard, robotic surgery has been reported as a minimally invasive alternative for gastric cancer, since it provides a more ergonomic position, wristed instruments that allow seven degrees of freedom, tremor filtering, the ability to scale motions, and 3D high definition stereoscopic vision that improves surgeon's dexterity when performing fine manipulations of tissue in a closed, fixed operating field.

Early studies using the robotic access date back from the second half of the past decade, mainly about partial resections and limited lymphadenectomy in patients in early stage disease. Song *et al.* published the first large series with 100 patients, 42 underwent extended lymphadenectomy. The average number of lymph nodes retrieved was 36.7, very close to the one obtained in the open operations^19^.

In a large series published by Yonsei University in South Korea, probably the center with the most experience in robotic gastrectomy, Woo *et al.* compared 591 operations by laparoscopy with 236 cases operated robotically. The operative time was longer in the second group. However, there was less blood loss, short-term surgical results were better and oncological results were similar^23^.

In a recent meta-analysis, Marano *et al.* analyzed 1,967 patients operated for gastric cancer (404 robotic, 718 open and 845 laparoscopic) and concluded that robotic gastrectomy reduces intraoperative blood loss and time of postoperative hospital stay compared with laparoscopy and laparotomy at a cost of longer surgical time and much more expensive costs. It also provides an adequate oncological lymphadenectomy^13^.

Hyung *et al*. conducted a multicenter prospective study with 434 patients comparing robotic (n=223) vs laparoscopic gastrectomy (n=211) for gastric cancer. The short-term outcomes showed similarity between both groups regarding overall complications, mortality, number of harvest lymph nodes, with longer operative time and higher costs in the robotic group^6^.

Barchi *et al*. described a technique for robotic digestive tract reconstruction after total gastrectomy in six patients with a laterolateral esophagojejunostomy with linear stapler. Although it was a small series, the authors demonstrated a safe technique, with no major complications and demands a relatively short time for its accomplishment, even when dealing with initial experience^1^.

## CONCLUSION

Minimally invasive gastrectomy with extended lymphadenectomy has been established as a technical alternative for early gastric cancer. Recent studies have shown that the laparoscopic approach is associated with lower rates of pain, blood loss, hospital stay, complications and mortality. The average number of removed lymph nodes is, at least, equal to open operations and although few studies have follow-up longer than five years, oncologic results appear to be similar. These data are likely to be confirmed by randomized trials results from Japan and South Korea. Concerning advanced gastric cancer, recent studies have demonstrated the safety and feasibility of the minimally invasive access. The mid-term oncological outcomes have encouraged surgeons to continue the development of this method. High cost is still a hindrance to the large-scale use of robotics. This advanced technological platform is nothing more than a sophisticated laparoscopic working tool for more complex procedures. It is an evolution in technology and improvement of instruments that may facilitate procedures such as radical gastrectomy. The knowledge gained from laparoscopic surgery should be used and incorporated into robotic surgery, which surely will result in an improvement of outcomes obtained in the minimally invasive treatment of gastric cancer.
